# Azacytidine and Decitabine Induce Gene-Specific and Non-Random DNA Demethylation in Human Cancer Cell Lines

**DOI:** 10.1371/journal.pone.0017388

**Published:** 2011-03-07

**Authors:** Sabine Hagemann, Oliver Heil, Frank Lyko, Bodo Brueckner

**Affiliations:** 1 Division of Epigenetics, Deutsches Krebsforschungszentrum, Heidelberg, Germany; 2 Genomics and Proteomics Core Facility, Deutsches Krebsforschungszentrum, Heidelberg, Germany; Roswell Park Cancer Institute, United States of America

## Abstract

The DNA methyltransferase inhibitors azacytidine and decitabine represent archetypal drugs for epigenetic cancer therapy. To characterize the demethylating activity of azacytidine and decitabine we treated colon cancer and leukemic cells with both drugs and used array-based DNA methylation analysis of more than 14,000 gene promoters. Additionally, drug-induced demethylation was compared to methylation patterns of isogenic colon cancer cells lacking both DNA methyltransferase 1 (DNMT1) and DNMT3B. We show that drug-induced demethylation patterns are highly specific, non-random and reproducible, indicating targeted remethylation of specific loci after replication. Correspondingly, we found that CG dinucleotides within CG islands became preferentially remethylated, indicating a role for DNA sequence context. We also identified a subset of genes that were never demethylated by drug treatment, either in colon cancer or in leukemic cell lines. These demethylation-resistant genes were enriched for Polycomb Repressive Complex 2 components in embryonic stem cells and for transcription factor binding motifs not present in demethylated genes. Our results provide detailed insights into the DNA methylation patterns induced by azacytidine and decitabine and suggest the involvement of complex regulatory mechanisms in drug-induced DNA demethylation.

## Introduction

Aberrant DNA methylation is a major hallmark of cancer [1], [2], [3]. In cancer cells, global hypomethylation is accompanied by hypermethylated and transcriptionally silenced tumor suppressor genes. These so-called epimutations contribute to the loss of proliferation control in cancer cells [4], [5], [6].

The maintenance of hypermethylation-induced epimutations requires the continuous activity of DNA methyltransferases (DNMTs) during cell division. Thus, inhibition of DNMTs has been successfully used in epigenetic cancer therapy to reverse epimutations and to reactivate epigenetically silenced tumor suppressor genes [7], [8], [9], [10]. The archetypal DNMT inhibitors 5-azacytidine (azacytidine, AZA) and 2′-deoxy-5-azacytidine (decitabine, DAC) have been approved for the treatment of myelodysplastic syndrome, a preleukemic bone marrow disorder. Despite their use in the clinic and in numerous preclinical studies, the knowledge of the mode of action of these drugs is still incomplete [11].

One of the major consistently observed cellular effects of azacytidine and decitabine is DNA demethylation. As nucleoside analogues, AZA and DAC are incorporated into replicating DNA where they can form covalent bonds with DNMTs [12],[13],[14]. This trapping of DNMTs leads to passive demethylation during DNA replication and cell division. Inhibition of DNA methylation by AZA and DAC has been successfully demonstrated at selected loci in various clinical studies [7], [9], [15].

Recently, the effects of AZA and DAC have also been investigated on the genomic level. Due to the limited availability of suitable tools for genome-wide methylation analysis, these studies were initially restricted to the analysis of drug-induced transcription changes. For example, gene expression profiling was used to analyze the effects of DAC on the gene expression pattern of HCT116 colon cancer cells and the results suggested that, besides gene activation of hypermethylated genes, transcriptional downregulation may be an important effect of DAC [16], [17]. More recently, Illumina GoldenGate arrays were used to directly characterize drug-induced DNA demethylation at 1,505 CG dinucleotides representing 807 cancer-related genes in myeloid leukemia cells [11]. However, due to the comparably low coverage of this array, the resulting data were not analyzed in detail and the molecular characteristics of DNA demethylation responses remained to be investigated.

In the present study, we used genome-scale Infinium analysis to systematically characterize the demethylation responses after AZA and DAC treatment in two human cancer cell lines. To this end we investigated methylation levels of more than 27,000 CG dinucleotides representing more than 14,000 genes [19] in HCT116 colon cancer cells and in HL-60 myeloid leukemia cells. Our results show that AZA and DAC demethylate CGs in non-CG islands more efficiently than those in CG islands (CGI). Moreover, treatment with AZA and DAC results in non-random and reproducible DNA demethylation patterns in HCT116 and HL-60 cells. Additionally, we identified a subset of CGs that is neither demethylated after drug-treatment nor in cells with extremely reduced levels of DNMT1 and no DNMT3B [20], [21]. Demethylation-resistant CGs are associated with genes preferentially bound by Polycomb Repressive Complex 2 (PRC2) components in ES cells and are enriched for transcription factor binding motifs not present in demethylated genes. These results unravel the patterns of DNA demethylation by AZA and DAC and suggest that drug-induced demethylation is regulated by defined molecular mechanisms.

## Materials and Methods

### Cell culture

Human HCT116 colon carcinoma cells and HCT116 double knockout (DKO) cells (DNMT1-/-; DNMT3B-/-) were kindly provided by Bert Vogelstein (July 2007) and cultured under standard conditions in McCoy's 5A medium supplemented with 5% L-glutamine and 10% FCS (Invitrogen). Identity of HCT116 cells was confirmed by DMSZ (Braunschweig, Germany; January 2008) using DNA profiling of eight short tandem repeats. HL60 cells were obtained from ATCC and cultured under standard conditions in RPMI medium (Sigma) supplemented with 5% L-glutamine and 10% FCS (Invitrogen). Fresh aliquots of all cell lines were used for experiments. To analyze the effects of 5-azacytidine (AZA) and 2′-deoxy-5-azacytidine (DAC), cells were cultivated in media supplemented with the compounds, at the concentrations indicated.

### Inhibitors

5 mM stock solutions of AZA (Sigma) and DAC (Calbiochem) were prepared by dissolving the substances in distilled H_2_O (GIBCO) and stored at −80°C. Immediately before treatment, stock solutions were diluted in cell culture medium to the concentrations indicated.

### Genomic DNA methylation analysis

Drug-treated cells were incubated with the indicated concentrations of AZA and DAC after a 24 h seeding period, and genomic DNA was prepared using the DNeasy Blood and Tissue Kit (Qiagen). Global genomic DNA methylation levels were determined by capillary electrophoresis as described previously [22].

### Array based DNA methylation analysis

Array-based gene-specific DNA methylation analysis was performed using Infinium HumanMethylation27 bead chip technology (Illumina) according to the manufacturer's instructions. Shortly, bisulfite treated genomic DNA was whole-genome amplified and hybridized to the HumanMethylation27 BeadChip. Oligomers, attached to two different bead types per interrogated locus, match either the unmethylated or the methylated state, enabling single-base extension and detection. The methylation status of a specific cytosine is indicated by average beta (AVB) values where 1 corresponds to full methylation and 0 to no methylation. Delta beta (DB) values were calculated by subtracting AVB values of treated or knockout cells from control AVB values. Biological replicates (see Figure S2) of each experiment were grouped and array probes with *P*≥0.05 were excluded from the analysis. Loci were scored as methylated if the AVB was greater than or equal to 0.2 [23]. The complete CG methylation data are available in the ArrayExpress database (www.ebi.ac.uk/arrayexpress). For a more detailed description of normalization and further calculations refer to Methods S1. Results were analyzed using Illumina's BeadStudio software, version 3.1.3.0 and with R, version 2.10.0 [24]. Specifically, following R packages were used for data analysis: graphics (boxplots), stats (kernel density estimates and statistical analyses), the limma package [25] for the construction of Venn diagrams and the lattice package [26] for the display of multivariate data (trellis plots).

### 454 DNA bisulfite sequencing

Deep DNA bisulfite sequencing of CGs of 4 genes (PIK3CG, Ells1, Aff2, NTRK3) was performed as described previously [27], [28]. For 454 sequencing, bisulfite-treated genomic DNA was amplified using sequence-specific primers containing treatment-specific barcodes and 454 linker sequences (Figure S7). 454 deep sequencing was performed by the DKFZ Genomics and Proteomics Core Facility.

### Analysis of transcription factor binding motifs

To identify enriched transcription factor binding motifs we used the online tool PScan [29] and 130 transcription factor-binding profiles (*H. sapiens*) from the JASPAR database [30]. The analysis of genes was focused on the region from -450 to +50, with respect to their transcription start site. To summarize the results of the PScan analysis, a heatmap displaying the natural logarithm of the *P* values was generated.

## Results

### Establishment of treatment conditions for AZA- and DAC-induced demethylation in HCT116 cells

**Figure 1:**
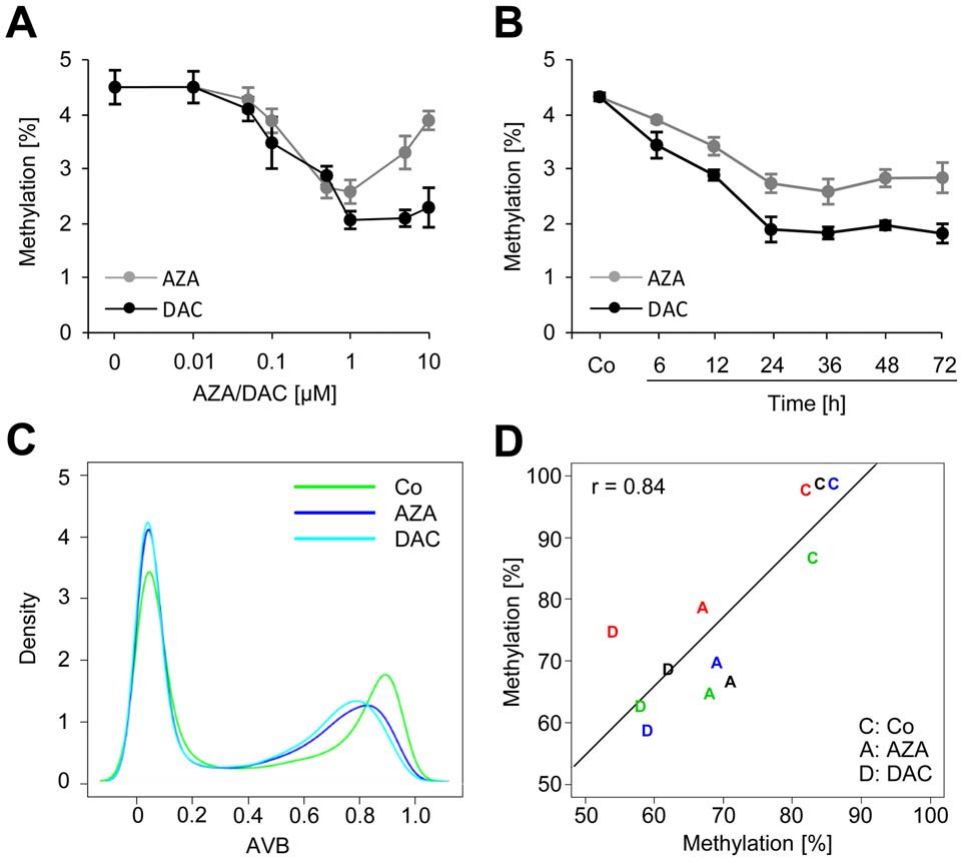
Genome-wide methylation analysis of HCT116 cells. A, Global methylation analysis (CE) of HCT116 cells treated with the indicated concentrations of AZA and DAC for 24 h. B, Time-course CE measurement of drug-induced demethylation using 1 µM AZA or DAC; Co, untreated HCT116 cells. C, Kernel density estimates of Infinium methylation data for untreated (Co) and drug-treated cells. D, Validation of Infinium methylation data using 454 bisulfite sequencing; methylation data of 4 CG loci, measured either by Infinium analysis or by 454 sequencing, correlate strongly (Pearson's correlation coefficient r  = 0.84); treatment with AZA and DAC is indicated by A and D, respectively; different loci are indicated by colors: PIK3CG (blue), NTRK3 (green), Ells1 (red), and AFF2 (black).

As a first step towards a systematic characterization of demethylation responses induced by azacytidine (AZA) and decitabine (DAC), we aimed to maximize demethylation efficiency, to minimize drug toxicity [31], [32] and to prevent remethylation as observed during long-term treatment [33]. To this end, we treated HCT116 cells with increasing drug concentrations (Figure 1A) and over various periods of time (Figure 1B) to determine global genomic methylation levels by capillary electrophoresis. The results clearly showed for both drugs that maximum demethylation was reached with concentrations of 1 µM after 24–36 h with DAC showing a 60% reduction and AZA showing a 50% reduction of global DNA methylation.

Since azanucleosides require DNA replication for their function, we analyzed whether the number of cells in S phase might be affected by drug treatment. HCT116 cells were treated with 0.1, 1, and 10 µM of AZA or DAC and cell cycle distribution was analyzed by FACS (Fluorescence Acitvated Cell Sorting). While the proportion of cells in S phase was increased after 24 h treatment with 0.1–10 µM AZA or DAC, only longer incubation times resulted in a decrease of replicating cells for several drug concentrations (Figure S1A). Furthermore, FACS analyses also revealed that only high drug concentrations (10 µM) resulted in a G2 phase arrest, which was accompanied by a reduction of cells in G1 phase (Figure S1B). Also, pronounced cell death was only observed with high drug concentrations, especially after treatment with 10 µM AZA, which indicates that AZA-treated cells stopped to replicate and died. These observations confirmed that demethylation should be analyzed after 24 h and at 1 µM drug concentration to exclude the confounding effects of drug toxicity.

### Array-based genome-wide DNA methylation analysis

To analyze DNA methylation patterns of HCT116 cells on a genome-wide scale we used Infinium methylation profiling to interrogate the methylation status of 27,578 CG dinucleotides representing 14,475 associated genes [11], [19]. Methylation of individual loci was determined by average beta (AVB) values that ranged from 0 (unmethylated) to 1 (completely methylated). Based on previous studies [19], only CGs that showed a decrease or increase in their AVB value (delta beta, DB) of at least 0.2 were used for the analysis of methylation changes. Biological replicates of HCT116 control cells and drug-treated cells showed a very high similarity (Figure S2A, B, C), confirming the technical robustness of the array and the high specificity of drug-induced methylation patterns.

Subsequent data analysis clearly revealed a bimodal distribution of CG dinucleotide methylation with a low-methylation peak (13,667 of 27,571 CGs) that was found at AVB values ranging from 0 to 0.2 and a high-methylation peak (8,222 of 27,571) that covered the interval from 0.8 to 1.0 (Figure 1C). As in untreated cells, the bimodal methylation distribution was also observed after treatment of cells with AZA and DAC (see Figure 1C). However, after drug treatment the high-methylation peak (AVB≥0.8 in control cells) was shifted to lower methylation values, indicating drug-induced demethylation.

To validate the methylation array results, we used highly quantitative 454 bisulfite sequencing [28] to analyze four strongly methylated CGs which became demethylated by drug-treatment in HCT116 cells. On average, we obtained about 300 reads per CG (see Figure S7). Correlation analysis of bisulfite sequencing data and array results (Figure 1D) showed a very good overall agreement of both methods (r = 0.84). These results demonstrate that the Infinium methylation array generates an accurate representation of the HCT116 methylation pattern in our experiments.

### Drug-induced demethylation patterns are non-random

**Figure 2:**
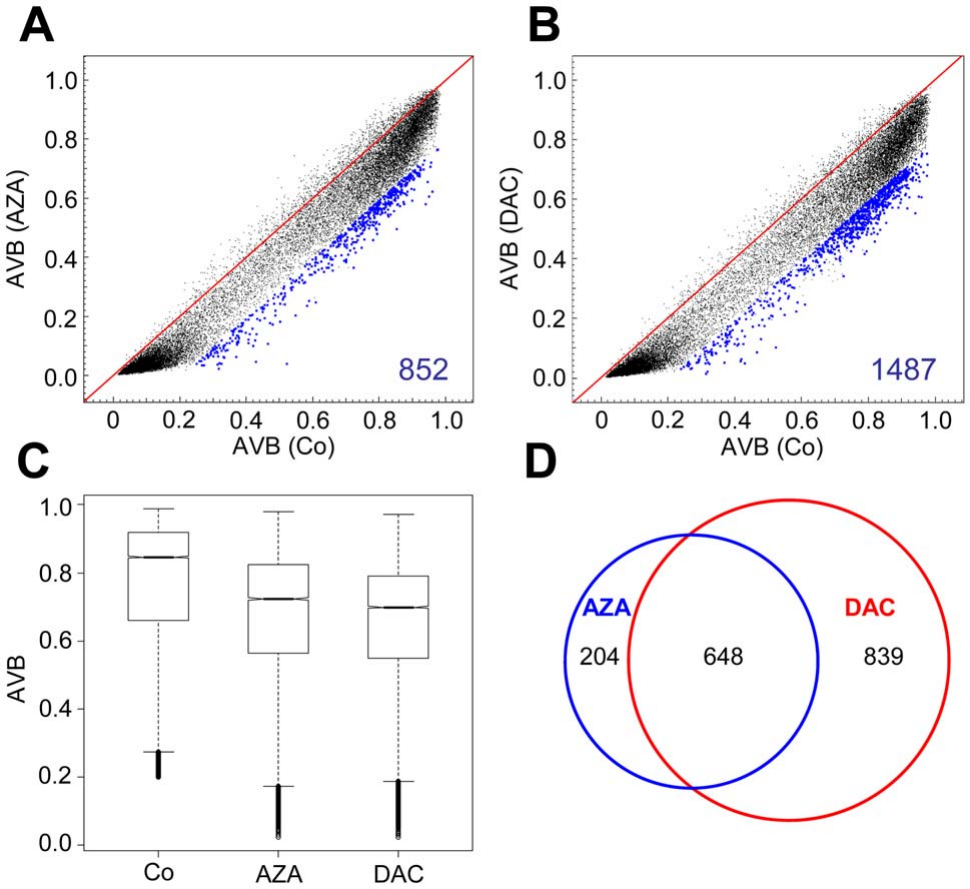
AZA and DAC induce non-random demethylation patterns in HCT116 cells. Methylation changes in HCT116 cells treated for 24 h with AZA (A) or DAC (B); blue dots and numbers represent demethylated CGs (DB≤−0.2). C, Boxplots show the distribution of methylated CGs in HCT116 control samples and cells treated with AZA or DAC; black lines denote medians, notches the standard errors, boxes the interquartile range, and whiskers the 2.5th and 97.5th percentiles. D, Venn diagrams indicate overlapping demethylated CGs in drug-treated cells.

Further analyses of array data showed that treatment with AZA resulted in demethylation (DB≤−0.2) of 6% (852 of 13,911) of the CGs methylated in control cells (Figure 2A). Showing an even higher efficacy, DAC treatment induced demethylation of 11% (1,487 of 13,911) of these CG sites (Figure 2B). A Wilcoxon rank sum test confirmed the significance of the difference between methylation in AZA- and DAC-treated cells (Figure 2C, *P*<2×10^−16^). The higher efficiency of DAC-mediated demethylation on the gene-specific level is consistent with our global methylation analysis in HCT116 cells (Figure 1A, B).

Based on the observation that demethylation patterns appeared to be surprisingly specific with high inter-replicate reproducibility, we wondered whether both drugs share commonly demethylated CG dinucleotides. To identify commonly demethylated CGs we grouped AZA and DAC replicates, respectively, and found a substantial overlap of CGs that were demethylated by both drugs (Figure 2D). This overlap was significantly greater than expected by random demethylation (*P*<2×10^−16^, Fisher's Exact Test), indicating that specific loci are preferentially demethylated by AZA and DAC. Despite the widespread demethylating activity of AZA and DAC, a substantial number of CG dinucleotides appeared resistant to drug-induced demethylation in HCT116 cells (Figure 2A, B).

### Resistance to drug-induced demethylation is mostly overcome in DNMT1; DNMT3B double knockout cells

**Figure 3:**
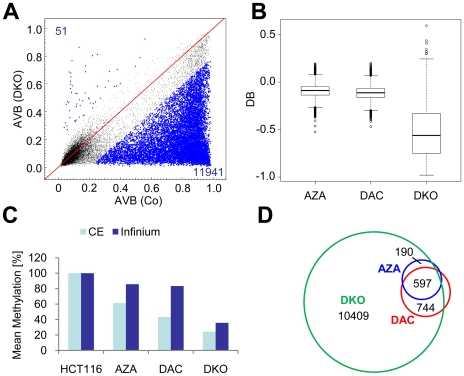
The majority of CGs resistant to drug-induced demethylation become demethylated in DKO cells. A, DKO cells show substantial differences to HCT116 cells in their methylation pattern; blue dots and numbers represent demethylated or hypermethylated CGs (DB≤−0.2 or ≥0.2). B, Boxplots show the demethylation (DB) in drug-treated HCT16 cells and DKO cells; black lines denote medians, notches the standard errors, boxes the interquartile range, and whiskers the 2.5th and 97.5th percentiles. C, Comparison of relative mean methylation of drug-treated cells and DKO cells as determined by global genomic methylation analysis (CE) and Infinium methylation analysis. D, Venn diagrams indicate overlapping demethylated CGs between drug-treated cells and DKO cells.

To further characterize the CGs resistant to drug-induced demethylation we obtained methylation profiles from HCT116 cells with strongly reduced levels of DNMT1 and complete loss of DNMT3B (DKO cells). Data analysis revealed pronounced demethylation in DKO cells with more than 85% of methylated CGs being demethylated (Figure 3A). DKO cells also showed the greatest degree of demethylation represented by median DB values of less than −0.55 relative to control cells (Figure 3B). In comparison, we observed significantly (*P*<2×10^−16^, Wilcoxon rank sum test) lower degrees of demethylation for AZA and DAC (Figure 3B).

We next compared the mean methylation level of gene-associated CGs derived from the Infinium array to global methylation measured by capillary electrophoresis (CE) (Figure 3C). In addition to Infinium methylation analysis, CE also interrogates CGs in repetitive elements which comprise the majority of methylated DNA in the human genome [34]. Interestingly, the degree of demethylation of whole genomic DNA was always higher than gene-specific demethylation. This suggests that CGs in repetitive elements became more efficiently demethylated than gene-associated CGs. When we compared demethylation patterns of drug-treated and knockout cell lines, we found that 92% of the CGs demethylated by AZA and 90% of the CGs demethylated by DAC were also demethylated in DKO cells (Figure 3D). However, our results indicate that drug-induced demethylation of specific genes is relatively inefficient when compared to the entire genome and to DNMT-deficient cells.

### Drug-induced demethylation of cancer-related and bona fide tumor suppressor genes

**Figure 4:**
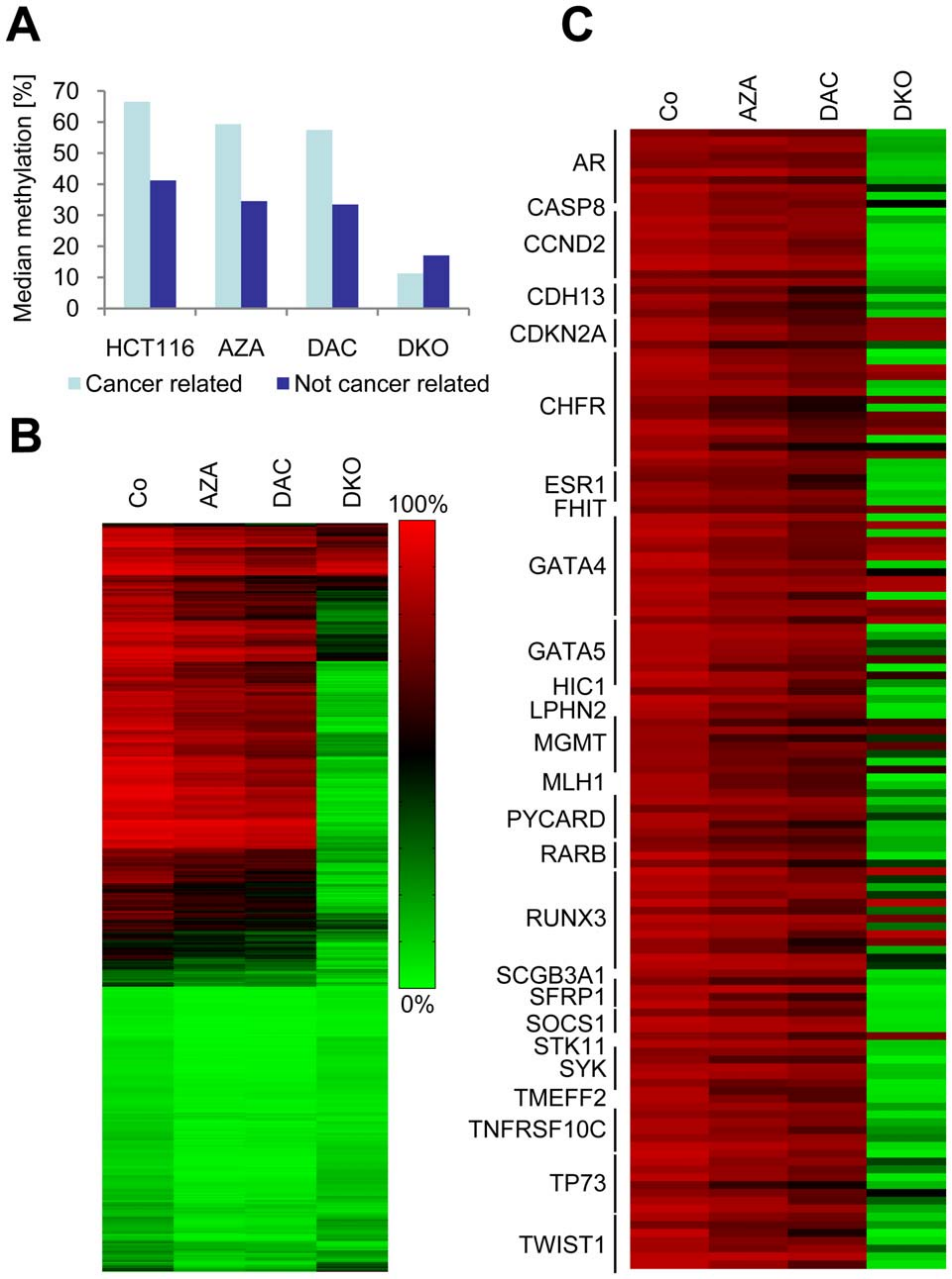
Hypermethylated cancer-associated genes and tumor suppressor genes are strongly demethylated in DKO cells. A, Median methylation levels of cancer-related and non-cancer-related CGs in untreated cells (Co), drug-treated cells (AZA, DAC) and DKO cells. B, Heatmap of CG methylation in cancer-associated genes. C, Heatmap of hypermethylated bona fide tumor suppressor-genes in drug-treated and DNMT knockout cells.

We next analyzed drug-induced demethylation in a panel of cancer-associated genes (adapted from the GoldenGate Methylation Cancer Panel I, Illumina). Out of 807 cancer-associated genes in this panel, 784 (represented by 2,125 CGs) were also present on the Infinium methylation chip. The analysis of our methylation data revealed that cancer-related genes were more highly methylated than non-cancer-related genes, which are present on the Infinium platform but not on GoldenGate (Figure 4A). Furthermore, cancer-related methylation was strongly reduced in DKO cells (Figure 4A).

A detailed analysis revealed that out of 2,125 cancer-associated CGs, a set of 906 CGs was hypermethylated (AVB≥0.8) in HCT116 control cells. DAC demethylated these hypermethylated cancer-associated genes with a similar efficiency as AZA (Figure 4B). Interestingly, we observed that almost all genes were strongly demethylated in DKO cells. Similar results were also obtained for a set of hypermethylated bona fide tumor suppressor genes (Figure 4C). These data further illustrate the ability of DAC and AZA to demethylate tumor suppressor genes, and suggest that drug-induced overall gene-specific demethylation is comparably weak.

### Non-CG islands and highly methylated CGs are preferentially demethylated

**Figure 5:**
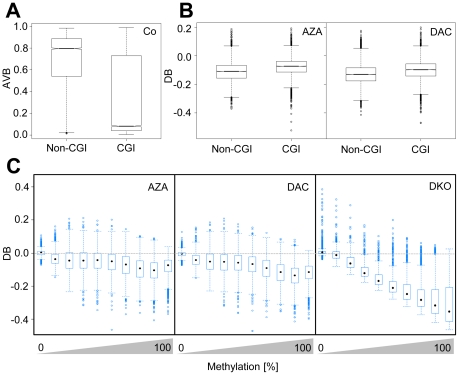
Highly methylated CGs and CGs outside of CG islands are preferentially demethylated in HCT116 cells. A, Boxplots indicate differences in methylation between CGIs and non-CGIs. B, Boxplots show demethylation efficiency indicated by DB values in AZA- and DAC-treated HCT116 cells dependent on CG association with CGIs. For A and B, black lines denote medians, notches the standard errors, boxes the interquartile range, and whiskers the 2.5th and 97.5th percentiles. C, Boxplots show demethylation efficiencies as a function of degree of CG-methylation in AZA- and DAC-treated HCT116 cells. Methylation levels were grouped in 10% intervals from 0 to 100% methylation; black marks denote medians, boxes the interquartile range, and whiskers the 2.5th and 97.5th percentiles.

To further refine our analysis, we distinguished between CGI and non-CGI-associated CGs. As expected [35], [36], CGs in non-CGIs were predominantly methylated (3,667 of 7,513, AVB≥0.8) whereas those in CGIs were mostly unmethylated (12,782 of 20,002, AVB≤0.2) (Figure 5A). In addition, we also observed a prominent fraction of highly methylated CGs that were associated with CGIs (4,527 of 20,002, AVB≥0.8), which is consistent with CGI hypermethylation in cancer [6]. Interestingly, our results show that both, AZA and DAC, demethylated a higher proportion of methylated CGs not located in CGIs. Specifically, in HCT116 cells, AZA demethylated 3.0% (219 of 7,224) of methylated CG dinucleotides in CGIs but 9.5% (633 of 6,687) methylated CGs in non-CGIs (Figure 5B); DAC demethylated 6.6% (474 of 7,224) of CG dinucleotides in CGIs but 15.2% (1,013 of 6,687) CGs in non-CGIs (Figure 5C). We therefore conclude that CG dinucleotides within CGIs became preferentially remethylated after drug-induced passive demethylation (*P*<2×10^−16^, Fisher's exact test).

To analyze whether demethylation efficiency is a function of the degree of CG methylation, we grouped CGs by their methylation level in 10 intervals from 10% to 100% methylation and determined to which extent CGs in different intervals were demethylated. Our results show that AZA- and DAC-induced demethylation was more efficient for highly methylated CGs (methylation intervals from 60% to 100% methylation). The significance of this effect was further illustrated by an analogous analysis of demethylation efficiency in DKO cells. Here, CGs of all methylation levels were demethylated equally well, as demonstrated by the constantly increasing distance of their medians to the baseline (Figure 5D). We therefore conclude that AZA and DAC preferentially lead to demethylation of highly methylated CG dinucleotides.

Since non-CGI-associated CGs show higher methylation levels than CGI-associated CGs (Figure 5A), we further analyzed if the differential methylation of both groups resulted in the observed difference in demethylation efficiency between CGI- and non-CGI-associated CGs (Figure 5B, C). To this end, we grouped CGs, according to their methylation level in untreated cells, in intervals of equal methylation and determined demethylation of CGs in CGIs and non-CGIs (Figures S3, S4). This analysis confirmed that for methylation levels greater than 50–60% in HCT116 cells (greater than 20–30% for HL60 cells, see below), CGs in non-CGIs become significantly more demethylated than CGs in CGIs.

### Drug-induced demethylation in myeloid leukemia cells

**Figure 6:**
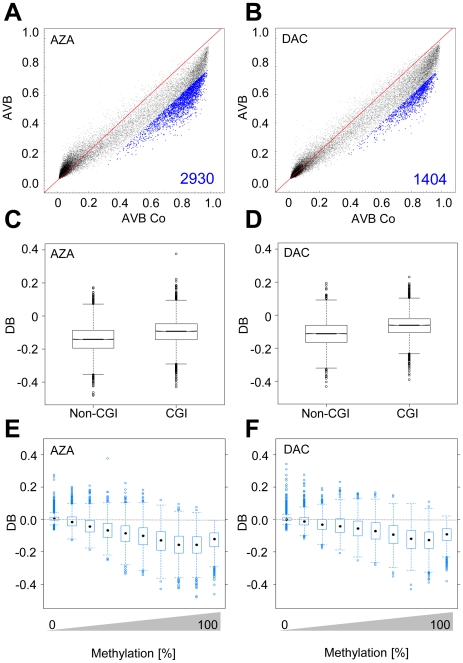
Drug-induced demethylation patterns in HL60 myeloid leukemia cells. Methylation changes in HL60 cells treated for 24 h with AZA (A) or DAC (B); blue dots and numbers represent demethylated CGs (DB≤−0.2). C, D, Boxplots show demethylation efficiency indicated by DB values in AZA- and DAC-treated HCT116 cells dependent on CG association with CGIs; black lines denote medians, notches the standard errors, boxes the interquartile range, and whiskers the 2.5th and 97.5th percentiles. Boxplots show demethylation efficiencies as a function of degree of CG methylation in E, AZA- and F, DAC-treated HL60 cells. Methylation levels were grouped in 10% intervals from 0 to 100% methylation; black marks denote medians, boxes the interquartile range, and whiskers the 2.5th and 97.5th percentiles.

We sought to confirm our previous findings in a model more closely related to the approved indication of DAC and AZA. To this end, we treated HL-60 myeloid leukemia cells for 24 h with drug concentrations that induced in the strongest demethylation response (0.5 µM DAC or AZA) and again obtained methylation profiles by Infinium analysis. The results showed that treatment with AZA resulted in strong demethylation (DB≤−0.2) of 16% (1,839 of 11,406) of the CGs methylated in control cells (Figure 6A). DAC treatment induced demethylation of 8% (941 of 11,406) of these CG sites (Figure 6B). Methylation levels between drug-treated cells differed significantly (*P*<2×10^−16^, Wilcoxon rank sum test), as described above for HCT116 cells, and we again observed a strong overlap of CGs demethylated by AZA and DAC (Figure S5E). The comparison of CGI-associated CGs and non-CGI-associated CGs showed that CGs in CGIs were less methylated than those in non-CGIs, which is in agreement with our findings in HCT116 cells. Also, as previously observed in HCT116 cells, AZA and DAC demethylated CGs in non-CGIs more efficiently than those in CGIs in HL60 cells (Figure 6C, D) (*P*<2×10^−16^, Wilcoxon rank sum test). Consistent with our findings in HCT116 cells, drug-induced demethylation was also more efficient for highly methylated CGs in HL60 cells (Figure 6E, F).

### Resistance to demethylation correlates with PRC2 occupancy and transcription factor binding

**Figure 7:**
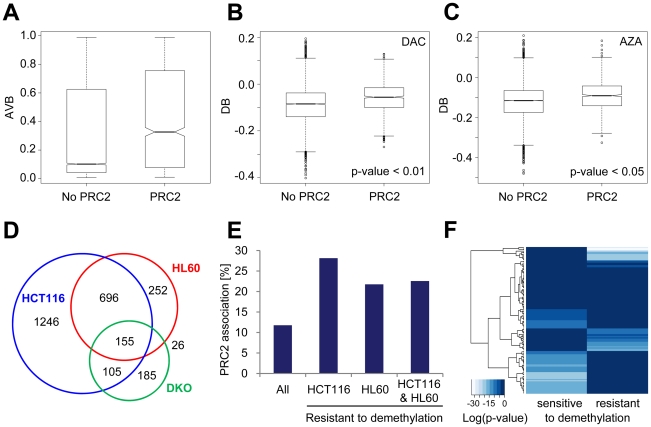
The chromatin environment may modify drug-induced demethylation efficiency. A, Boxplots show the distribution of CG methylation in PRC2-associated CGs and non-PRC2-associated CGs in HL60 cells. B, C, Boxplots show demethylation efficiency indicated by DB values in AZA- and in DAC-treated HL60 cells dependent on association with PRC2 components. For A, B, and C black lines denote medians, notches the standard errors, boxes the interquartile range, and whiskers the 2.5th and 97.5th percentiles. D, Venn diagrams indicate CGs which did not become demethylated (AVB≥0.8) in drug treated HCT116 and HL60 cells and in DKO cells, respectively. E, Percentage of demethylation-resistant CGs associated with PRC2 components in HCT116 and HL60 cells, and for overlapping CGs of both cell lines (HCT116 & HL60). F, Significance of enrichment of transcription factor binding sites in genes with demethylation-resistant CGs in HCT116 and HL60 cells and with CGs that became demethylated by AZA and DAC in HCT116 and HL60 cells (sensitive to demethylation). Heatmap columns represent log (*P* values) for enrichment of 130 transcription factors.

We finally considered potential molecular mechanisms which may modulate drug-induced demethylation efficiency. Previous studies have suggested that specific binding of Polycomb complexes (PRC2) may induce hypermethylation of gene promoters during tumorigenesis [37], [38]. Accordingly, PRC2-associated regions may be predisposed to rapid remethylation after replication. Thus, we assigned genome-wide association data for SUZ12, EED, and H3K27 trimethylation [39] to the corresponding CGs on the Infinium chip. Our analysis revealed that CGs associated with the interrogated PRC2 components show higher median methylation than CGs not associated with PRC2 (Figure 7A). Interestingly, PRC2-associated CGs were significantly more resistant to demethylation by AZA and DAC (Figure 7B, C).

To refine this analysis, we subsequently focused on demethylation-resistant CGs. We identified a set of 1129 gene-associated CGs which were resistant to demethylation by AZA and DAC in HL60 cells (AVB≥0.8). Interestingly, 75% of these CGs were also resistant to drug-induced demethylation in HCT116 cells (Figure 7D). A detailed analysis showed that PRC2 components were strongly enriched at promoter regions of CGs resistant to demethylation in HCT116 and HL60 cells, as well as in overlapping resistant CGs of both cell lines (Figure 7E). To identify further distinguishing features of demethylation-sensitive and -resistant CGs, we also analyzed if genes harbouring demethylation-resistant CGs are characterized by different sets of transcription factor binding motifs compared to genes that become demethylated. Using the software tool Pscan [29], we analysed the presence of binding sites for 130 transcription factors in 644 genes (851 CGs) that were resistant to demethylation in HCT16 and HL-60 cells and 121 genes (128 CGs) that became demethylated in both cell lines after drug treatment. The analysis revealed that demethylation-sensitive and -resistant CGs are associated with genes that show a complementary enrichment of transcription factor binding sites (Figure 7F, Figure S8). Interestingly, the corresponding transcription factors also belong to different transcription factor families (see Figure S9). For example, binding sites of Forkhead box (Fox) transcription factors are enriched in demethylation sensitive genes whereas basic Helix-Loop-Helix (bHLH) transcription factor binding sites are enriched in demethylation resistant genes. These results confirm the notion that specific molecular mechanisms, based on sequence context and chromatin configuration, are involved in the regulation of drug-induced DNA demethylation.

## Discussion

The role of DNA methylation in tumor cell biology has been systematically analyzed in HCT116 cells and DNMT knockout cells in two previous studies [16], [17]. These studies utilized the indirect approach of pharmacological unmasking [40] to identify methylated genes through changes in their transcriptional profile. Interestingly, recent data show that AZA and DAC induce different sets of genes with only little overlap [41]. This finding is consistent with previously published data indicating that both compounds are metabolized differently and thereby induce different effects on cellular viability [42]. In the present study, we have now directly analyzed the demethylation pattern of HCT116 and HL60 cells after drug-treatment by interrogating the methylation status on the genome-scale. In contrast to the above mentioned differential effects of AZA and DAC on cell viability and gene expression, our data demonstrate a substantial overlap of genes demethylated by both drugs. This is likely due to the fact that both compounds are ultimately incorporated into DNA as identical metabolites (i.e. 5-aza-2′-deoxycytidine-5′-triphosphate) [18], where they function as DNMT inhibitors. However, presuming a random mechanism of DNA demethylation, the strong overlap of CGs demethylated by AZA and DAC was surprisingly high. In addition, a high reproducibility of demethylation patterns was also observed for biological replicates of drug-treated cells (Figure S5A, B, C, D). In agreement with the currently accepted model of passive demethylation, one would expect that every round of replication leads to a reduction of DNA methylation by half, in the absence of functional maintenance methylation activity. Thus, our observation indicates that specific loci remain demethylated after replication whereas others appear resistant because they become preferentially remethylated by DNMTs, leading to a non-random pattern of demethylation.

The resistance to drug-induced demethylation was overcome for most CGs in DKO cells with minimal amounts of DNMT1 and no DNMT3B activity. These cells showed more demethylated loci than any of the drug-treated cells which is in agreement with a previous study that analyzed gene expression changes in these cells [16]. Although drug treatment led to depletion of both DNMT1 and DNMT3B (Figure S6), drug-induced demethylation was much less efficient than demethylation in DKO cells. While extended treatment with AZA and DAC might lead to stronger demethylation, our results suggest that the demethylation effects observed in DKO cells cannot be achieved by the use of DAC or AZA.

However, a significant number of CGs was found to be resistant to demethylation even in strongly demethylated DKO cells. About 33% of these CGs were also resistant to drug-induced demethylation in HCT116 and HL60 cells (155 CGs), which again indicates that specific CGs became more rapidly remethylated after replication than other CGs. This preferential remethylation of specific loci suggests the involvement of factors which may modulate the cellular methylation efficiency after replication.

Importantly, array-based analysis also revealed that the efficiency of demethylation depends on the degree of methylation of CG dinucleotides as well as on their localization within or outside of CGIs. We observed that AZA and DAC preferentially led to demethylation of CGs not located in CGIs, whereas CGI-associated CGs became preferentially remethylated. Interestingly, 78% of the demethylation-resistant CGs in HCT116 and HL60 cells were located in CGIs, which confirms our notion about the demethylation-resistance of CGIs. This further indicates that a subset of CGs, conserved across different cell lines, might be a target of specific regulatory mechanisms.

The resistance of methylated CG dinucleotides in CGIs to drug-induced demethylation in HCT116 colon cancer and HL60 leukemic cells might partly be explained by the higher densities of CG nucleotides in CGIs and associated proteins. Therefore, also the chromatin surrounding the hypermethylated CGIs in HCT116 cells could possibly contribute to the preferential remethylation of these regions. Consistent with this notion, it has previously been demonstrated that genes associated with PRC2 components in ES cells show increased levels of DNA methylation in cancer cells [37], [38]. Correspondingly, we observed a significant enrichment of PRC2 components at these demethylation-resistant loci, which may mediate rapid remethylation of associated CG dinucleotides after replication. In line with this hypothesis, EZH2 has been shown to interact with DNMTs and thus may recruit these enzymes to specific loci [38]. Especially interesting is our finding of the complementary enrichment of transcription factor binding motifs, which belong to different transcription factor families, in resistant and demethylated loci. We found that binding sites of transcription factors of the Forkhead box (Fox) family are enriched in demethylated but not in demethylation-resistant genes. Intriguingly, Fox transcription factors have been demonstrated to induce transcriptional competence [43] and implicated in the formation of unmethylated gene-specific regions in ES cells [44]. Thus, differences in transcription factor binding may also account for the observed differences in drug-induced demethylation efficiency at specific loci. This is in line with a recent report [45] that demonstrated the enrichment of defined sequence motifs in CGIs resistant to de novo methylation. Correspondingly, binding of transcription factors might interfere with maintenance methylation at defined regions of the newly synthesized DNA strand during replication and thereby mediate non-random demethylation at specific loci. Additionally, other factors with sequence-specific binding motifs such as MeCP2 might also recruit transcriptional repressor complexes to specific loci and thereby indirectly mediate rapid remethylation at demethylation-resistant CGs [46].

The involvement of complex regulatory mechanisms in drug-induced DNA demethylation may also explain the observed low demethylation efficiency at many hypermethylated loci during epigenetic cancer therapy [11]. Interestingly, our data show that long-term depletion of both DNMT1 and DNMT3B, as observed in DKO cells, may overcome demethylation-resistance at many loci which supports the development of specific and non-toxic inhibitors for clinical improvement of epigenetic therapy.

## Supporting Information

Methods S1 **Statistical analysis of Infinium methylation array data.**
(PDF)

Figure S1 **FACS analysis of HCT116 cells treated with 0.1, 1, and 10 µM of AZA or DAC.**
(TIF)

Figure S2 **Kernel density distribution of AVB values from biological replicates.** Co, untreated HCT116 cells; AZA, azacytidine treated HCT116 cells; DAC, decitabine treated HCT11 cells.
(TIF)

Figure S3 **Difference in demethylation efficiency between CGI- and non-CGI-associated CGs (DAC, left panel; AZA, right panel) in HCT116 cells.** Difference is significant (P<0.05, pairwise Wilcoxon rank sum tests) for methylation levels greater than 0.5.
(TIF)

Figure S4 **Difference in demethylation efficiency between CGI- and non-CGI-associated CGs (DAC, left panel; AZA, right panel) in HL-60 cells.** Difference is significant (P<0.05, pairwise Wilcoxon rank sum tests) for methylation levels greater than 0.2.
(TIF)

Figure S5 **Venn diagrams show high reproducibility of demethylation patterns for biological replicates of drug-treated cells.**
(TIF)

Figure S6 **Western blot of DNMT1 and DNMT3B protein levels after 24 h drug treatment with the indicated concentrations.** Beta actin was used as a loading control.
(TIF)

Figure S7 **Primer sequences used for 454 sequencing.** Adapter sequences are indicated in red and sample-specific bar codes in green. Number of sequencing reads for each CG is indicated in the bottom panel.
(TIF)

Figure S8 **PScan enrichment analysis of 130 transcription factor binding sites in demethylated and non-demethylated genes (see main text for details).** Heatmap columns represent log (*P* values) for enrichment of 130 transcription factors.
(TIF)

Figure S9 **PScan enrichment analysis of transcription factor binding families in demethylated and non-demethylated genes (see main text for details).**
(TIF)
