# Supplementary material for: Azacytidine and Decitabine Induce Gene-Specific and Non-Random DNA Demethylation in Human Cancer Cell Lines
Source: PLoS One. 2011 Mar 7;6(3):e17388. doi: 10.1371/journal.pone.0017388 (PMC3049766; doi:10.1371/journal.pone.0017388)
Supplement: Methods S1 — Statistical analysis of Infinium methylation array data. (PDF) [file pone.0017388.s001.pdf]

**Statistical analysis of Infinium methylation array data**

The complete Infinium raw data were quantile normalized. Quantile normalization [1] does not assume any specific distribution, however, the shape of the underlying distribution should be the same for all samples. As this is not the case for the green and the red channel in the Infinium methylation assay, we quantile normalized these two channels separately. As methylation indicator of a given target either the green or the red channel is used according to the annotation provided by Illumina. For each CG on the chip two probeIDs (A and B) are utilized. The beta-value [2] is calculated using the green (grn) channel as beta = (grn.B) / (grn.B + grn.A + 100) or using the red channel as beta = (red.B) / (red.B + red.A + 100). The methylation as described by the beta value ranges from 0 (unmethylated) to 1 (fully methylated). For each beta value two variables (grn.A/B or red.A/B) are quantified and considered as independent. Therefore, aggregating beads and calculating mean values over groups of samples has been done for each of the two variables separately. We calculated the means, standard deviations and p-values of grn.A/B or red.A/B for each probeID in every sample. All beads of one probeID of all samples of a group were used to calculate the mean values, standard deviations, detection p-values and beta values of a given probeID in the group. Because in general one of the two variables cannot be distinguished from background, the detection p-value is defined as the minimum of the two separate p-values of the two variables, where each p-value is the result of testing (Mann-Whitney-U-test) against the negative beads of each channel. This minimum can still be large ($p > 0.05$) which is interpreted as a missing signal. As we measure two variables to calculate the beta value we apply the Benjamini-Hochberg [3] correction before choosing the minimum. These adjusted detection p-values give the probability for the type I error of rejecting the true null hypothesis "the mean beta values of the probeID and the negative beads are equal". For differential methylation analysis the difference of beta values was calculated using the mean beta values of the two groups. The statistical significance of each difference was obtained by calculating the two p-values of the two independent variables accounted for each beta value, again on the bead level as described above. As we cannot assume a normal distribution in the different aggregated data sets we used the two-sample Wilcoxon test (Mann-Whitney-U-test) two calculate p-values. These p-values give the probability for the type I error of rejecting the true null hypothesis "the mean beta values of the two groups are equal". For global statements about overall methylation effects the p-values have been adjusted using the Benjamini-Hochberg [3] method.

References

[1] Probe Level Quantile Normalization of High Density Oligonucleotide Array Data, Ben Bolstad, Division of Biostatistics, University of California, Berkeley December 2001.

[2] BeadStudio Methylation Module v3.2, User Guide, Illumina.

[3] Y. Benjamini and Y. Hochberg (1995). Controlling the false discovery rate:a practical and powerful approach to multiple testing. Journal of the Royal Statistical Society B, Vol. 57, 289–300.
