# Supplementary figures and images for: Azacytidine and Decitabine Induce Gene-Specific and Non-Random DNA Demethylation in Human Cancer Cell Lines
Source: PLoS One. 2011 Mar 7;6(3):e17388. doi: 10.1371/journal.pone.0017388 (PMC3049766; doi:10.1371/journal.pone.0017388)

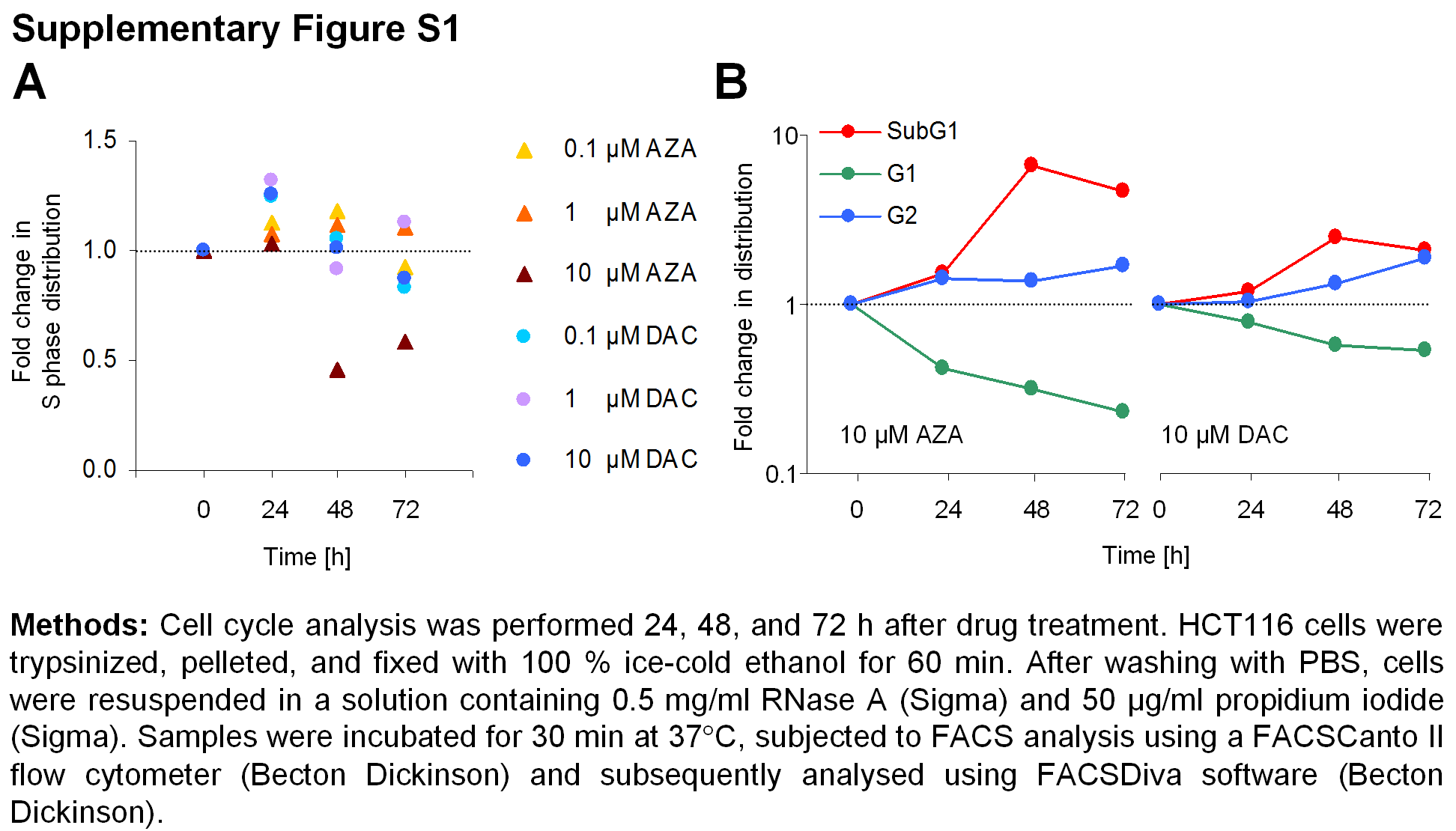

Supplement: Figure S1 — FACS analysis of HCT116 cells treated with 0.1, 1, and 10 µM of AZA or DAC. (TIF) [file pone.0017388.s002.tif]

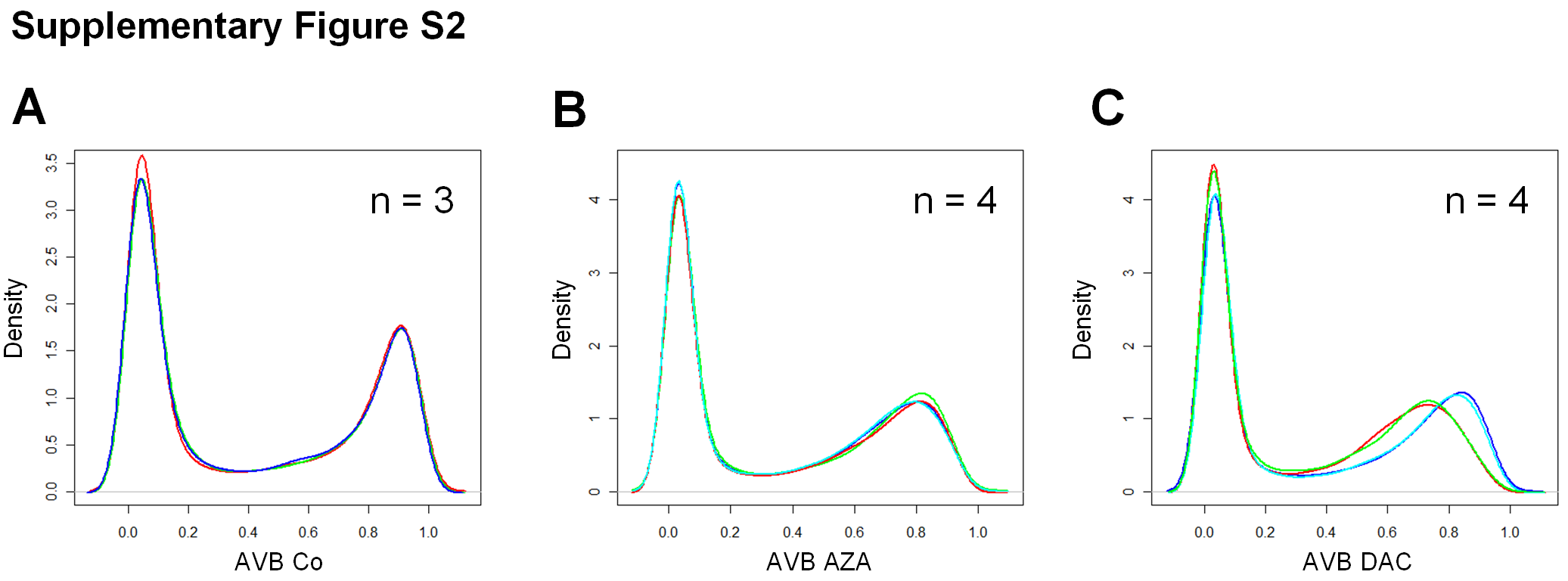

Supplement: Figure S2 — Kernel density distribution of AVB values from biological replicates. Co, untreated HCT116 cells; AZA, azacytidine treated HCT116 cells; DAC, decitabine treated HCT11 cells. (TIF) [file pone.0017388.s003.tif]

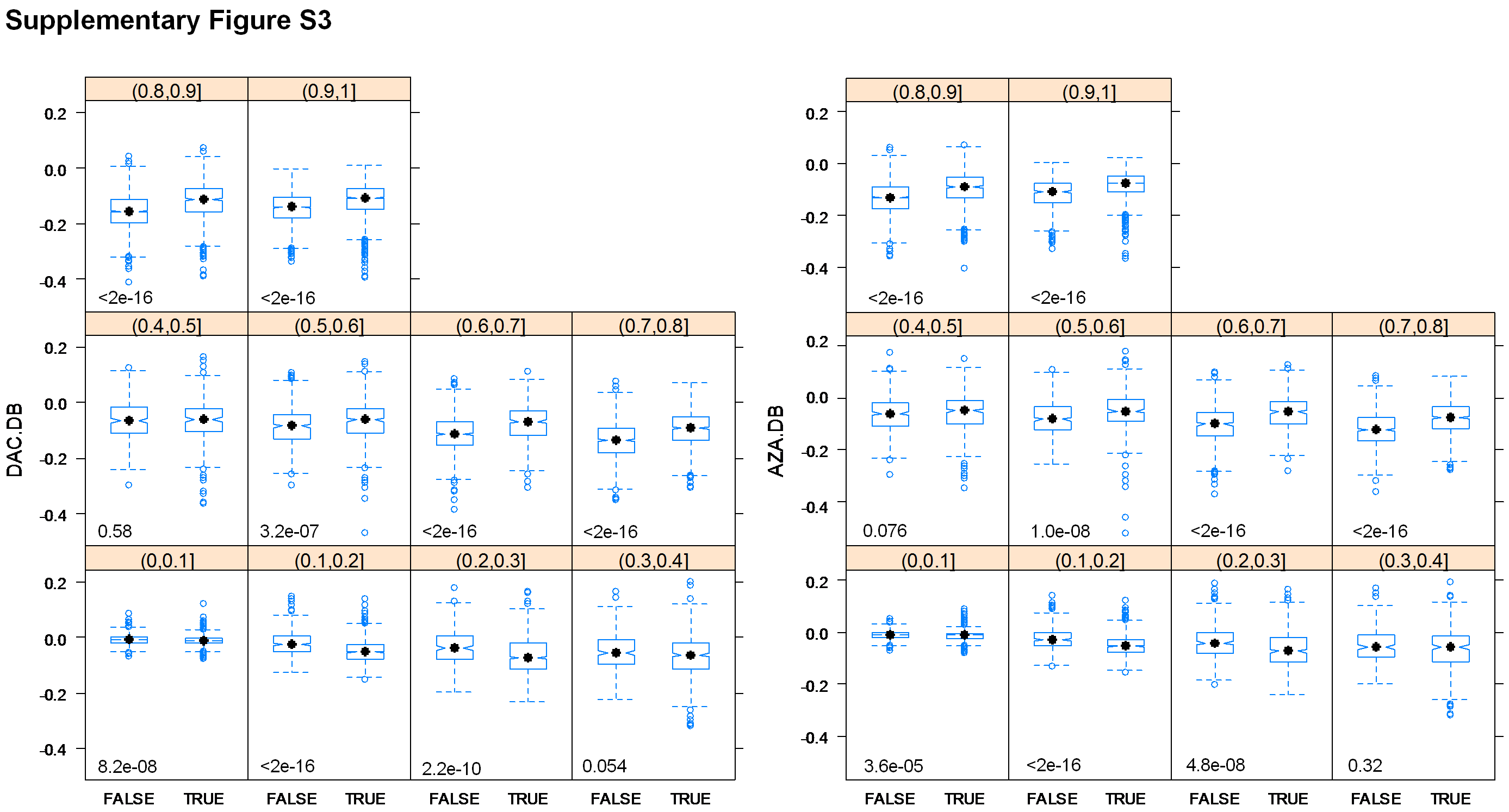

Supplement: Figure S3 — Difference in demethylation efficiency between CGI- and non-CGI-associated CGs (DAC, left panel; AZA, right panel) in HCT116 cells. Difference is significant (P<0.05, pairwise Wilcoxon rank sum tests) for methylation levels greater than 0.5. (TIF) [file pone.0017388.s004.tif]

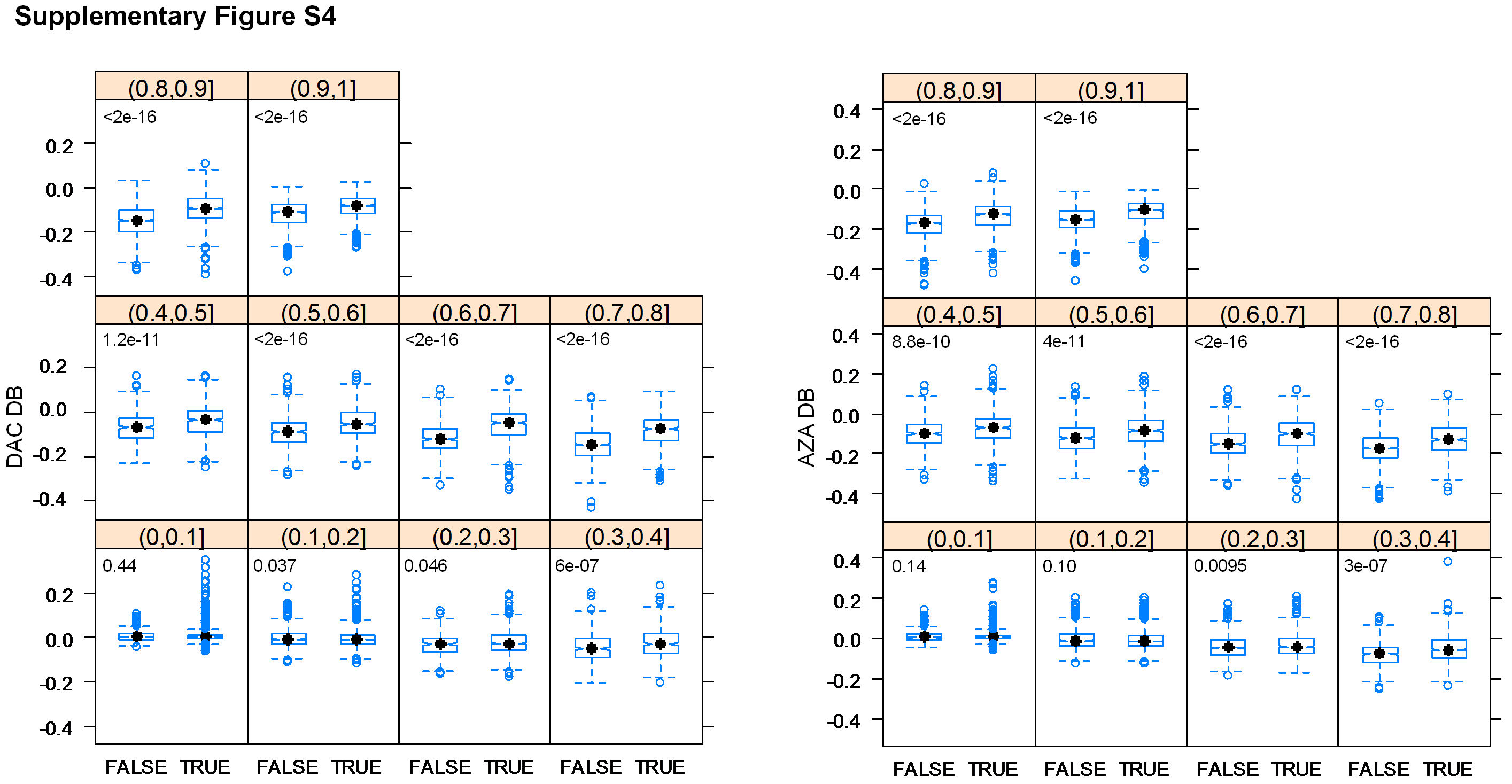

Supplement: Figure S4 — Difference in demethylation efficiency between CGI- and non-CGI-associated CGs (DAC, left panel; AZA, right panel) in HL-60 cells. Difference is significant (P<0.05, pairwise Wilcoxon rank sum tests) for methylation levels greater than 0.2. (TIF) [file pone.0017388.s005.tif]

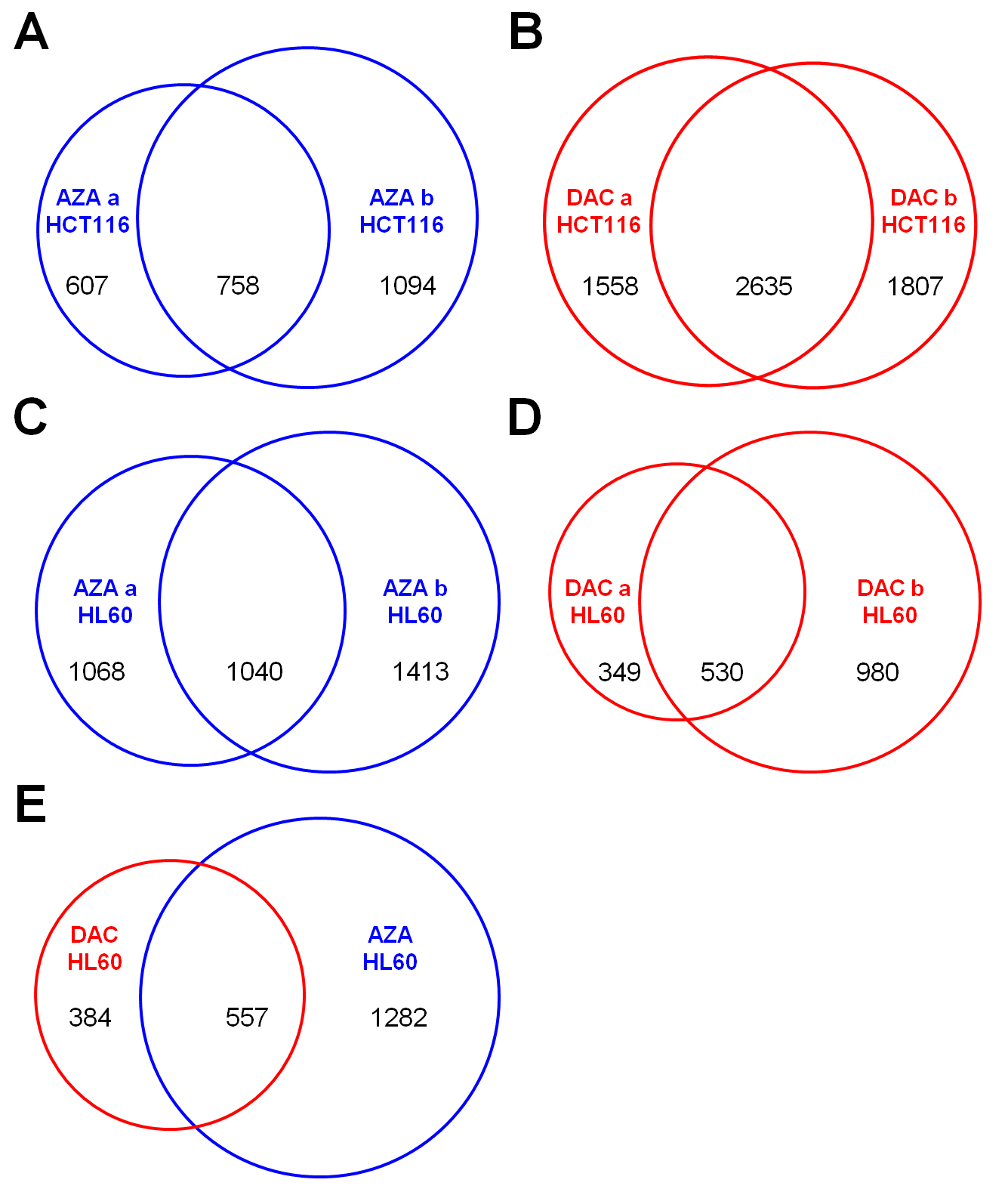

Supplement: Figure S5 — Venn diagrams show high reproducibility of demethylation patterns for biological replicates of drug-treated cells. (TIF) [file pone.0017388.s006.tif]

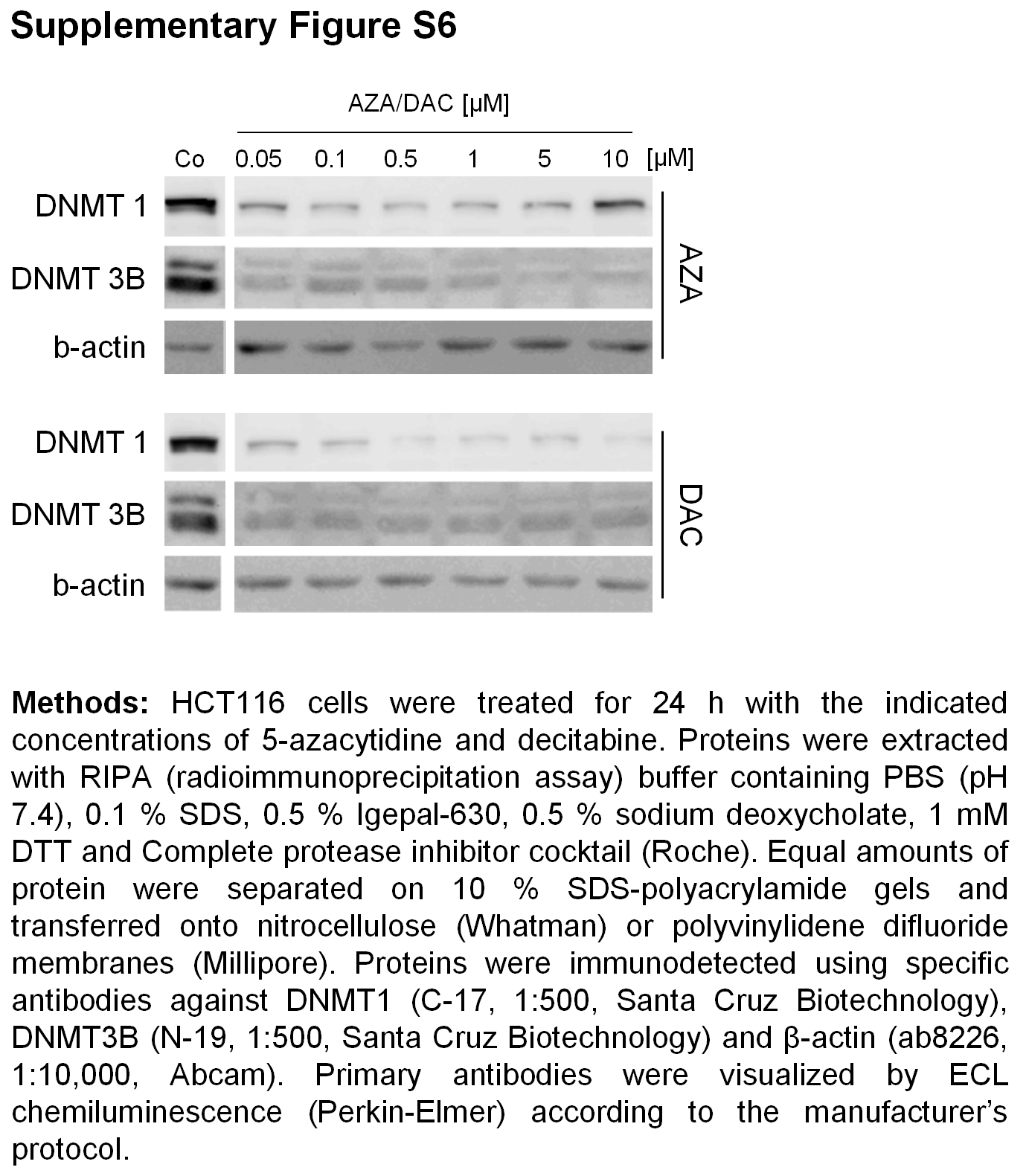

Supplement: Figure S6 — Western blot of DNMT1 and DNMT3B protein levels after 24 h drug treatment with the indicated concentrations. Beta actin was used as a loading control. (TIF) [file pone.0017388.s007.tif]

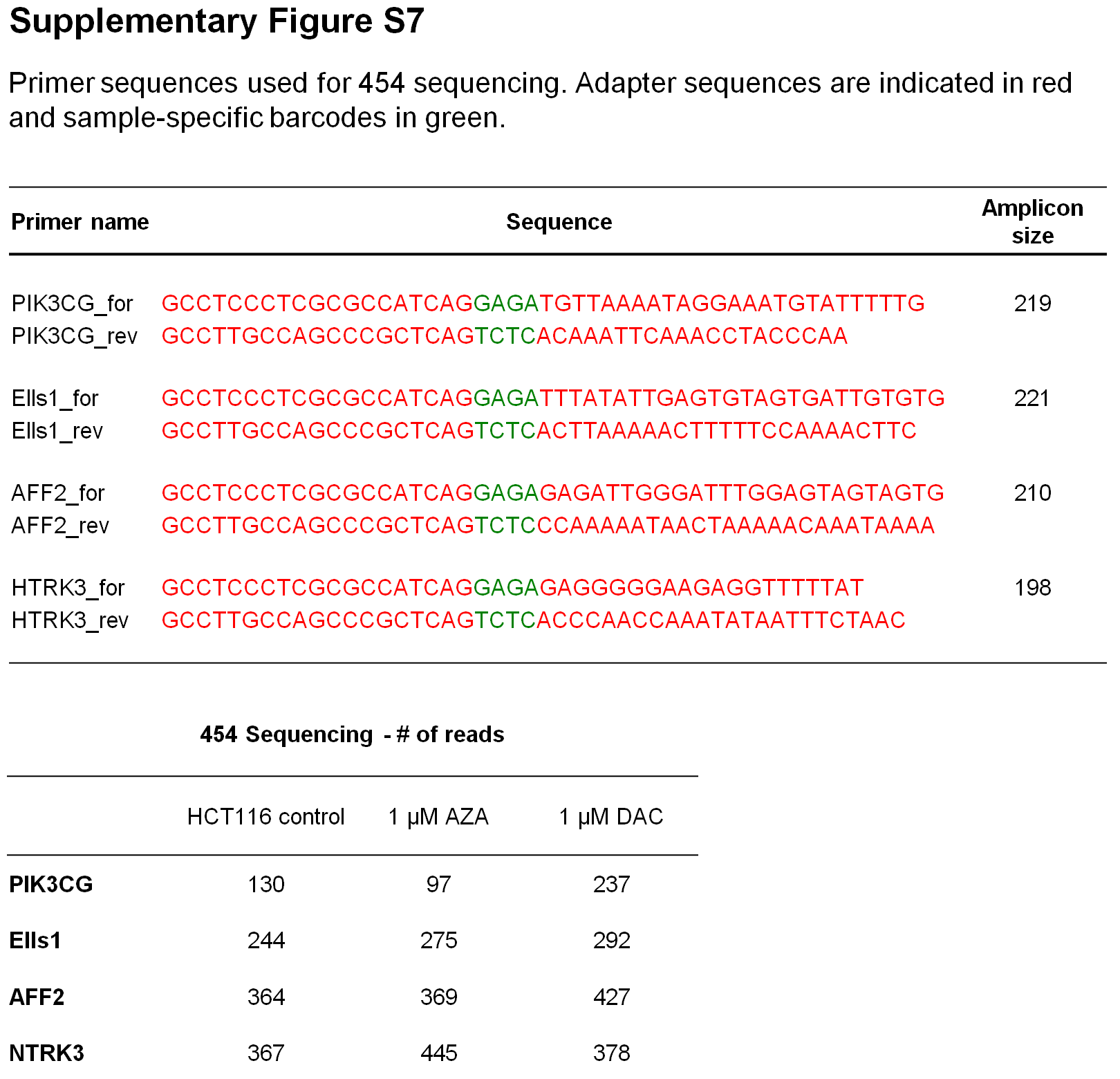

Supplement: Figure S7 — Primer sequences used for 454 sequencing. Adapter sequences are indicated in red and sample-specific bar codes in green. Number of sequencing reads for each CG is indicated in the bottom panel. (TIF) [file pone.0017388.s008.tif]

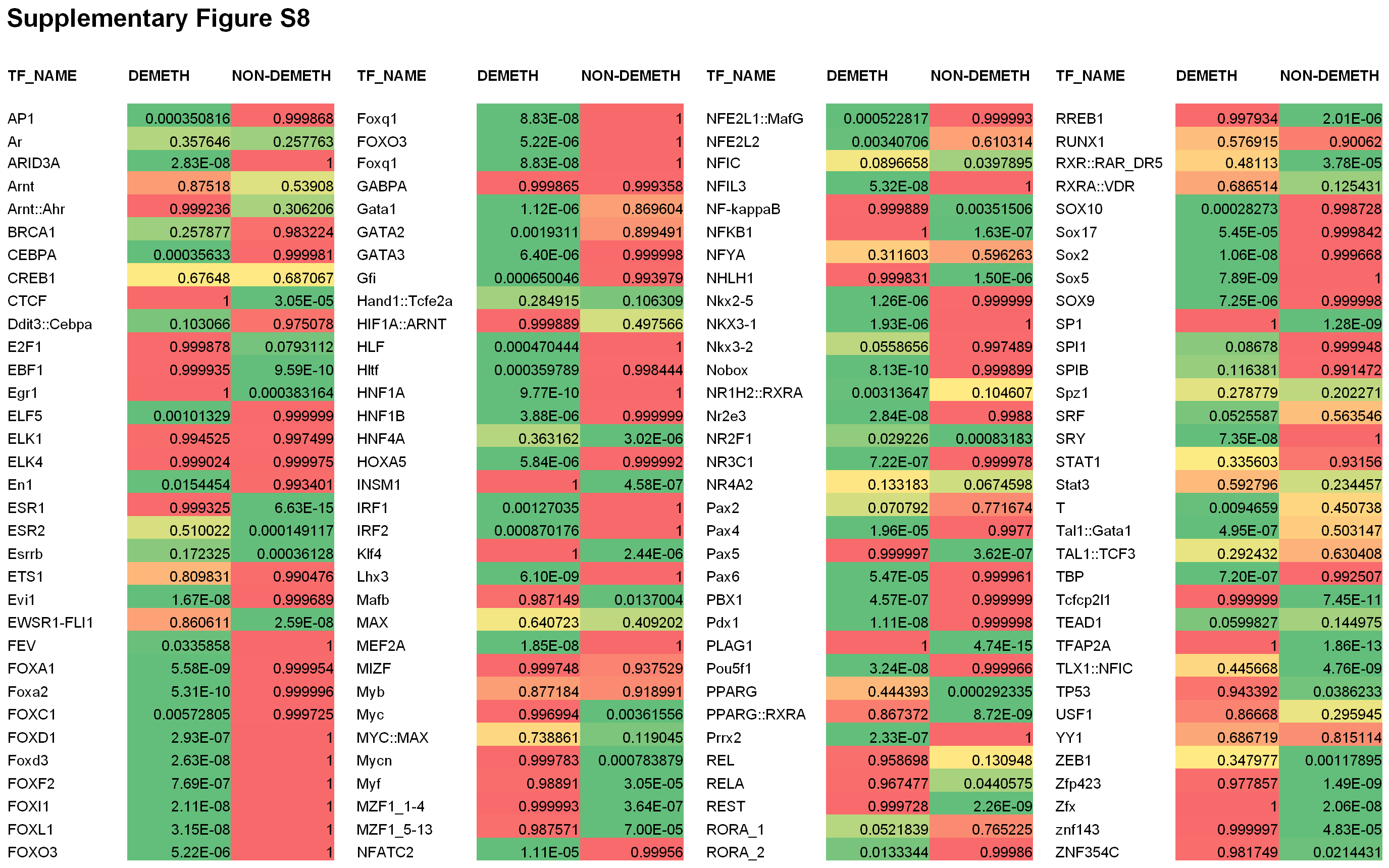

Supplement: Figure S8 — PScan enrichment analysis of 130 transcription factor binding sites in demethylated and non-demethylated genes (see main text for details). Heatmap columns represent log (P values) for enrichment of 130 transcription factors. (TIF) [file pone.0017388.s009.tif]

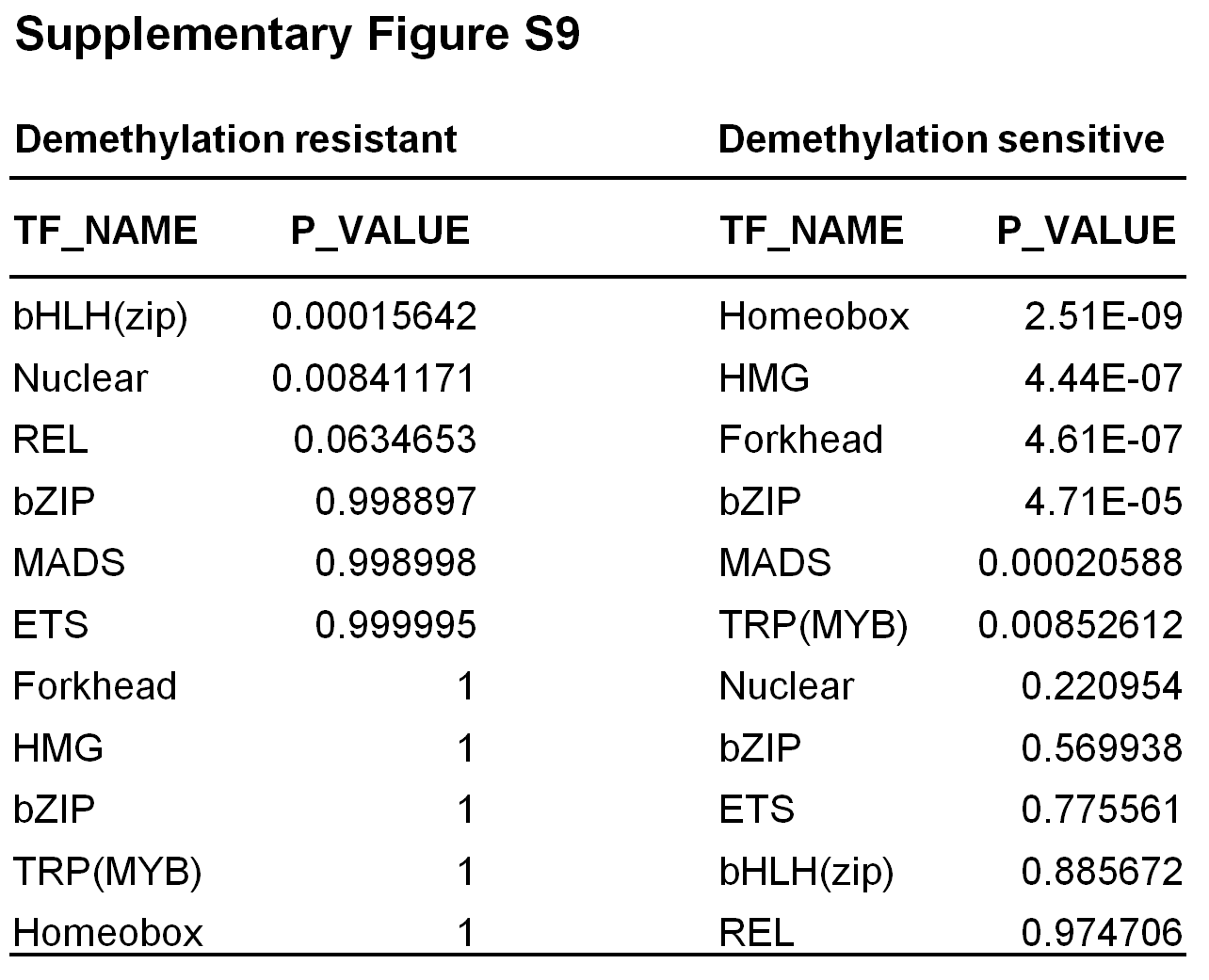

Supplement: Figure S9 — PScan enrichment analysis of transcription factor binding families in demethylated and non-demethylated genes (see main text for details). (TIF) [file pone.0017388.s010.tif]
